# Deletions of neuraminidase and resistance to oseltamivir may be a consequence of restricted receptor specificity in recent H3N2 influenza viruses

**DOI:** 10.1186/1743-422X-6-22

**Published:** 2009-02-14

**Authors:** Shelly Gulati, David F Smith, Gillian M Air

**Affiliations:** 1 Department of Biochemistry & Molecular Biology, University of Oklahoma Health Sciences Center, Oklahoma City, OK 73104, USA; 2 Department of Biochemistry, Emory University School of Medicine, Atlanta, GA 30322, USA

## Abstract

**Background:**

Influenza viruses attach to cells via sialic acid receptors. The viral neuraminidase (NA) is needed to remove sialic acids so that newly budded virions can disperse. Known mechanisms of resistance to NA inhibitors include mutations in the inhibitor binding site, or mutations in the hemagglutinin that reduce avidity for sialic acid and therefore reduce the requirement for NA activity.

**Results:**

Influenza H3N2 isolates A/Oklahoma/323/03 (Fujian-like), A/Oklahoma/1992/05 (California-like), and A/Oklahoma/309/06 (Wisconsin-like) lost NA activity on passage in MDCK cells due to internal deletions in the NA-coding RNA segment. The viruses grow efficiently in MDCK cells despite diminished NA activity. The full length NA enzyme activity is sensitive to oseltamivir but replication of A/Oklahoma/323/03 and A/Oklahoma/309/06 in MDCK cells was resistant to this inhibitor, indicating that NA is not essential for replication. There was no change in HA activity or sequence after the NA activity was lost but the three viruses show distinct, quite restricted patterns of receptor specificity by Glycan Array analysis. Extensive predicted secondary structure in RNA segment 6 that codes for NA suggests the deletions are generated by polymerase skipping over base-paired stem regions. In general the NA deletions were not carried into subsequent passages, and we were unable to plaque-purify virus with a deleted NA RNA segment.

**Conclusion:**

H3N2 viruses from 2003 to the present have reduced requirement for NA when passaged in MDCK cells and are resistant to NA inhibitors, possibly by a novel mechanism of narrow receptor specificity such that virus particles do not self-aggregate. These viruses delete internal regions of the NA RNA during passage and are resistant to oseltamivir. However, deletions are independently generated at each passage, suggesting that virus with a full length NA RNA segment initiates the first round of infection.

## Background

Influenza viruses have two membrane bound surface glycoproteins, hemagglutinin (HA) and neuraminidase (NA). HA is involved in virus attachment to cell surface receptors and mediates entry of the virus into the cell by a membrane fusion process. NA is required for virus release. The enzyme catalyses cleavage of the α-ketosidic linkage between a terminal sialic acid and an adjacent sugar residue. The removal of sialic acid from the carbohydrate moiety of newly synthesized hemagglutinin and neuraminidase is necessary to prevent aggregation of the virions at the cell surface [1,2]. This receptor-destroying role assumes similar specificity of HA and NA, and there are several reports describing reciprocal changes in HA affinity and NA activity [3-5]. However, the specificities of HA and NA are not always matched [2,6,7]. We previously showed that a Fujian-like virus, A/Oklahoma/323/03, does not elute from red blood cells by its own NA activity or even with *Vibrio cholerae *sialidase [8], indicating that NA activity does not cleave the receptor bound by the HA. Efficient growth of A/OK/323/03 in tissue culture suggested that either the non-cleavable receptor of red blood cells is not present, or the virus is not dependent on receptor destroying activity. We have now shown that A/Oklahoma/323/03 and several subsequent isolates accumulate large internal deletions of the neuraminidase coding sequence. The resulting loss in NA activity has no detrimental effect of growth of the viruses in MDCK cells.

## Results

We used four H3N2 influenza viruses that were isolated in primary rhesus monkey kidney (RMK) cells from throat swabs in the winters of 2003, 2005, 2006 and 2008. All the isolates grew to high yield (HA titer = 16–64) in the first passage when transferred to Madin-Darby canine kidney (MDCK) cells. The HA and NA sequences showed that A/Oklahoma/323/03 is similar to the A/Fujian/411/02 vaccine strain [8] while A/Oklahoma/1992/05, A/Oklahoma/309/06 and A/Oklahoma/483/08 are closely related to H3N2 vaccine strains A/California/7/04, A/Wisconsin/67/05 and A/Brisbane/10/07 respectively.

### NA activity of A/OK/323/03 decreased on passage

**Figure 1:**
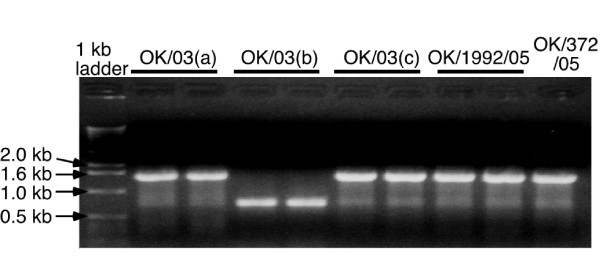
**Deletion of the NA coding RNA segment of A/OK/323/03**. NA-specific RT-PCR products of H3N2 virus stocks from MDCK cells separated on a 1% agarose gel stained with ethidium bromide. Lane1,1 kb ladder; Lanes 2–6 are duplicate loadings of RT-PCR products from three different stocks of A/OK/323/03; Lane 7–9 are two different 2005 isolates. The full-length NA RNA segment is 1467 nucleotides.

**Figure 2:**
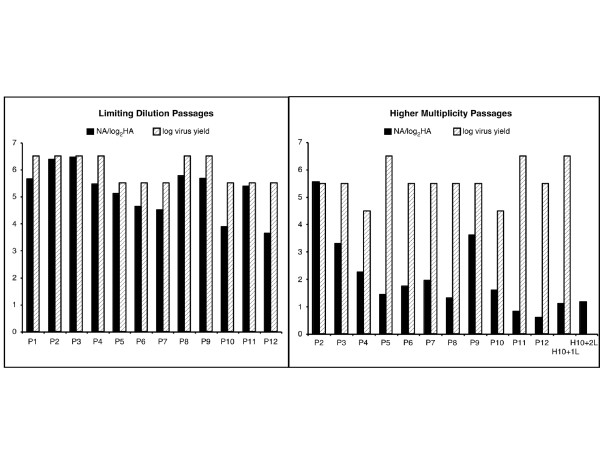
**NA activity of A/OK/323/03 was reduced during passage at moi 0.01 but not at limiting dilution**. The virus yield at each passage (TCIU) is compared to NA activity expressed as NA/HA ratio during limiting dilution passages (left) or moi 0.01 passages (right). Measurements were made on the first well for 0.01 moi passages and on the last well that showed infection for the limiting dilution passages. For comparison at the same scale, we plotted the ratio NA/(log_2_HA) and log(TCIU).

A/OK/323/03 grows robustly in MDCK cells (10^6^–10^7 ^TCIU per 10^5 ^cells) but several times we noticed a loss in NA activity as the virus was passaged. We ran a PCR reaction of RNA segment 6 using NA specific primers complementary to the non coding region at the 3' and 5' ends and found that the PCR product from a virus stock that had lost NA activity was about half the size of the full-length NA gene segment (Figure 1). To determine when the deletions occurred, we passaged the original virus stock from RMK cells in MDCK cells, as 10-fold serial dilutions in 6-well plates, under two different conditions of infection. One was limiting dilution, using 1 μl of the last well that showed infection to infect the first well of the next passage each time. In the other condition, we used 1 μl of the first well of the dilution series to infect the first well of the next passage (an average of 1000 TCIU per 10^5 ^cells, moi 0.01). We measured HA and NA activities and the infectious virus yield (TCIU) at each passage, P_1 _to P_12_. There was no significant decrease in the HA activity or in the titer of released virus during the passages under either condition but, starting from P_3_, the NA activity was reduced under the higher multiplicity conditions and did not recover even when the virus was passaged under limiting dilution following the high multiplicity passages (Figure 2). Under limiting dilution conditions there was little decrease in NA activity, as measured by NA/HA ratio. There was no significant difference in virus growth under the two conditions; for passages 3 through 12, the average HA log_2 _titer was 5.7 ± 1.1 under limiting dilution and 5.3 ± 1.1 at the higher multiplicity of infection (moi), while the average log(TCIU) was 5.9 ± 0.5 under limiting dilution and 5.6 ± 0.7 at the higher moi.

We passaged A/Oklahoma/1992/05 five times under the same conditions and found ~50% decrease in NA activity but no change in HA titer or in the titer of released virus during the passages. With this virus, the decrease in NA activity occurred under both limiting dilution and moi 0.01 conditions.

### Deletions in the NA gene

**Figure 3:**
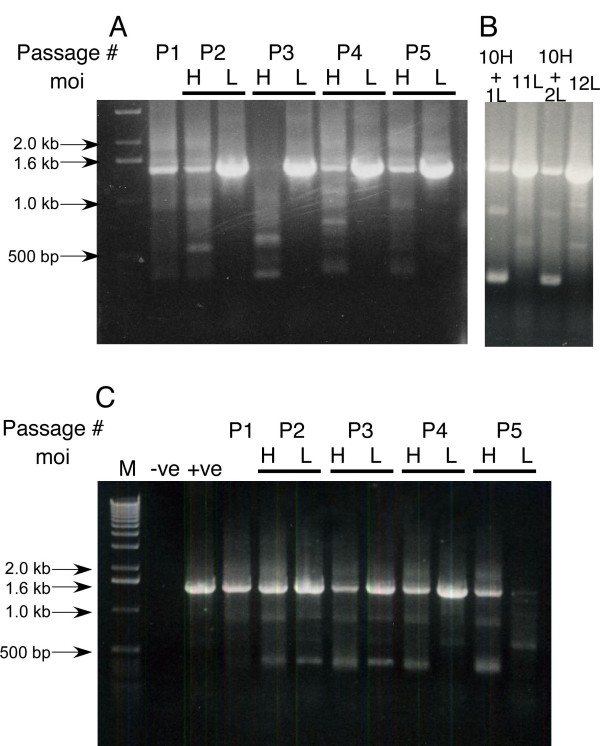
**Deletions in NA RNA as viruses were passaged at limiting dilution or at moi of 0.01**. RT-PCR products of NA genes from different passages amplified from viral cDNA were separated on a 1% agarose gel and stained with ethidium bromide. A and B: A/OK/323/03. A. Lane1, 1 kb ladder; lanes 2–6 are P_1_-P_5_; H = higher multiplicity (moi = 0.01) passages; L = limiting dilution passages. Limiting dilution passages retained the full length NA band, but passages at higher moi had little full length band and showed faster migrating bands resulting from internal deletions of the NA RNA segment. B. PCR products after further passages. 10H+1L is 10 passages at 0.01 moi followed by one passage at limiting dilution; 11L is limiting dilution passage 11; 10H+2L is 10 passages at high moi followed by two at limiting dilution; 12L is limiting dilution passage 12. C. A/OK/1992/05. Lanes are the same as panel A with the addition of negative (no RNA) and positive controls. Deletions are seen at P2 and higher but there is only minor decrease in full length NA at the higher multiplicity compared to limiting dilution.

We carried out reverse transcriptase PCR (RT-PCR) on the medium from each passage of A/OK/323/03 using NA specific primers designed from the 3' and 5' ends of the NA gene segments. The size and sequence of the PCR product from limiting dilution passages was the 1467 nucleotides expected for full-length NA, with no mutations compared to the parental virus. However, with higher multiplicity infection the NA gene segment gave short PCR products (Figure 3A). In most passages there was still a trace of full-length NA PCR product and so after P_10 _we passaged the higher multiplicity virus stock twice at limiting dilution (H10+1L, H10+2L) to see if the virus reverted back to its original NA length and activity. There was no significant increase in NA activity (Figure 2) despite a higher proportion of PCR product at the full-length position on the gel. After both limiting dilution passages there was a prominent PCR product at ~300 bp (Figure 3B). We passaged A/OK/1992/05 under the same conditions of limiting dilution and 0.01 moi and again saw short PCR products, but, in accord with the results for NA activity, there was no consistent difference between limiting dilution and higher multiplicity passages (Figure 3C).

**Figure 4:**
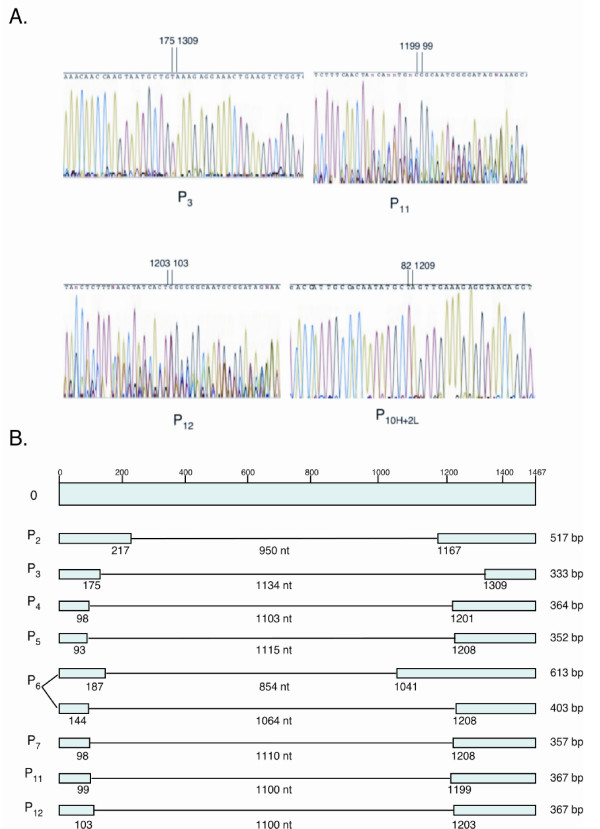
**Subgenomic NA segments showed different internal deletions but all retained the 5' and 3' ends**. A. Sequence chromatograms showing the junctions in some deleted NA segments of A/OK/323/03. The numbers indicate the junction nucleotides. B. Schematic showing examples of deletions of the NA coding region of A/OK/323/03. In all sequences, the 5' and 3' ends are retained. Lines represent internal deletions and the number of nucleotides deleted is indicated. The junction nucleotides are noted in each case. Two unrelated deletions are shown for Passage 6. Other sequences were derived from NA but the junctions were unclear due to mixed products in the gel bands.

Sequences of bands cut from the gels from P_2 _to P_12 _of A/OK/323/03 showed internal deletions from the normal length of 1467 nucleotides down to ~300 bp (Figure 4). We sequenced short PCR bands that appeared to be well separated at each passage. As expected, some gel bands contained mixed sequences that were uninterpretable. However, all the PCR products that yielded clear sequence represented NA-specific RNA fragments, each resulting from a single internal deletion within the NA gene. Both ends of the NA RNA were retained in all the deleted NA products, with 82 nucleotides or more at the 5' end and 158 or more nucleotides at the 3' end. We saw no carryover of deleted products from one passage to the next; even when PCR products appeared the same size, the sequences showed different junctions in each passage (Figure 4). However, when the high multiplicity P_10 _was passaged twice at limiting dilution, a fragment with a deletion of 1126 nucleotides (82/1209) was retained through both limiting dilution passages (Table 1).

**Table 1:** Single internal deletions in the NA genes of H3N2 viruses isolated from 2003 to 2008.

Virus	Passage No.^1^	First block 1 to ...	Second block ... to 1467	Length of fragment
Fujian-like virus				

OK/323/03	P7Ld1	181	1194	454

	P10H+1Ld1	82	1209	

	P10H+2Ld1	82	1209	341

	P13Ld1	261	1181	547

	P14Hd1	73	1207	333

				

California-like virus				

OK/1992/05	P2Hd1	51	1148	370

	P2Ld1	145	1244	368

	P3Ld1	145	1244	368

	P3Hd1	125^2^	1301	320

				

Wisconsin-like viruses				

OK/1472/06	P1d2	221	1166	522

OK/51/06	P1d1	40	1244	263

				

Brisbane-like viruses				

OK/5545/07	P1d1	231	1308	390

OK/1806/08	P1d1	145	1210	402

OK/483/08	P1d1	145	1261	351

^1^PCR products are numbered from larger to smaller. P2Hd1 is the larger deleted product seen at passage 2 (P2) under higher (H) moi. P7Ld1 was from the 7^th ^passage under limiting dilution (L).

^2 ^29 nucleotides in between are unidentified

Deleted NA genes were obtained from A/Oklahoma/1992/05 starting in the second passage in MDCK cells under either limiting dilution or 0.01 moi; there was more full length NA under limiting dilution conditions but not the dramatic difference compared to higher moi seen with A/OK/323/03. At limiting dilution, a deleted PCR product of A/OK/1992/05 NA at P3 had the identical 1097 nucleotide deletion to one at P2 (junction 145/1244; Table 1). A/Oklahoma/309/06 and A/Oklahoma/483/08 showed short NA-specific PCR products even at P1 in MDCK cells (Table 1). In contrast to the single internal deletions seen so far, A/Oklahoma/309/06 yielded mosaic structures containing multiple junctions and repeated sequences. Some were mixtures in which the junctions were unclear, but one sequence was unambiguous (Table 2). We investigated another Wisconsin-like virus, A/OK/1472/06 and found a similar mosaic structure in a deleted NA RNA (Table 2). To determine if these were indeed from single RNAs we cloned A/Oklahoma/309/06 P1 PCR products into pBluescript. The resulting clones showed the same mosaic sequences as found in the direct sequencing of PCR products.

**Table 2:** Mosaic fragments of the NA gene

Virus	5' end cRNA	Joined to	Joined to	Joined to	Joined to
After 1 passage in MDCK cells					

A/OK/1472/06	1 – 63	1 – 81	1290 – 1467		

A/OK/309/06	1 – 108	194 – 324	120 – 190	1227 – 1467	

					

After 3 passages in the presence of oseltamivir					

A/OK/323/03	1 – 191	1210 – 1287	1301 – 1411	1419 – 1467	

A/OK/309/06	1 – 70	1 – 74	130 – 204	1253 – 1290	1310 – 1447

We plaque purified viruses from several stocks containing deleted NA gene segments but were unable to isolate any virus that lacked a full length NA RNA.

We attempted to follow the generation of the deletions by real-time PCR with SYBR green detection using primer sets that amplified (a) the conserved 3' and 5' end sequences or (b) the middle portion that was deleted. We hoped to see an increase in "a" product as the ends came closer together and a decrease in "b" product as the NA became deleted, either during a single cycle experiment or over multiple cycles, but there was no significant change in the ratios at 6 hr, 18 hr or 72 hr. A better experiment would have used a primer set within the retained 5' or 3' regions rather than spanning the whole segment, but no suitable primers were identified by the ABI software, probably because of the high A (or U) content (33.4%) of influenza mRNA (or vRNA) strands.

### A/OK/323/03 and A/Oklahoma/309/06 are sensitive to Tamiflu in the enzymatic assay but resistant in MDCK culture

**Figure 5:**
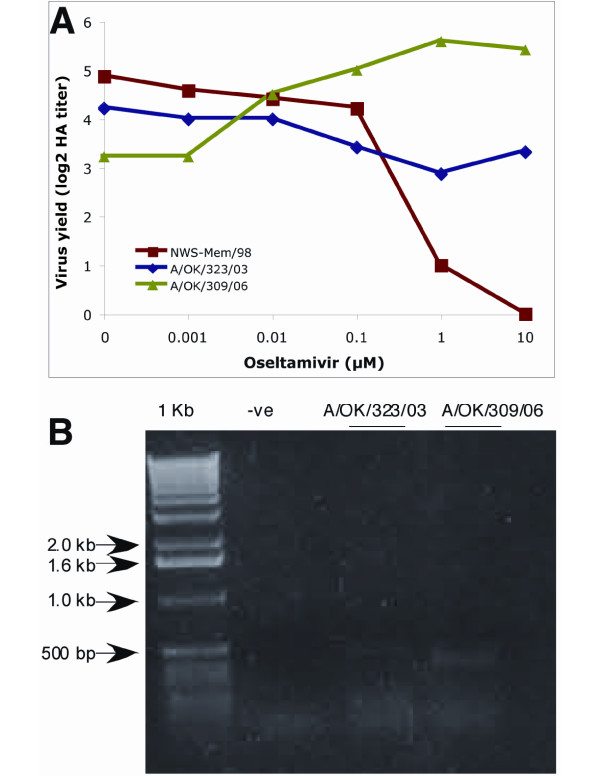
**A. A/OK/323/03 and A/OK/309/06 can grow in the presence of oseltamivir**. The results shown are the virus yield, expressed as HA titer, for the average of two different virus doses grown in increasing concentrations of oseltamivir carboxylate. NWS-Mem/98 (H1N9) is included as a control virus that is sensitive to oseltamivir inhibition. B. PCR products of NA gene after growth of A/OK/323/03 and A/OK/309/06 in oseltamivir. No full-length NA gene segment was visible but small fragments appear as diffuse bands less than 500 bp in length.

The high level of NA deletions suggests that little or no NA activity is required for multicycle replication of A/OK/323/03 in MDCK cells. We tested this by growing viruses in the presence of oseltamivir. In our standard fluorescence NA assay, oseltamivir carboxylate inhibited NA activity of A/OK/323/03 with an IC_50 _of 8 nM and A/OK/309/06 inhibition was similar (IC_50 _6 nM). We inoculated MDCK cells with serial dilutions of A/OK/323/03 that had been grown under strictly limiting dilution conditions and so had full-length NA gene segment and high NA activity, and grew the virus for one passage in the presence and absence of oseltamivir carboxylate (a kind gift from Dr. Warren Kati, Abbott). Ten fold dilutions of oseltamivir starting at 10 μM were added to the infection medium immediately after virus adsorption, the plates incubated at 37°C for 3 days, and virus yield measured by HA titration. The results are shown in Figure 5. Replication of a control virus, NWS-Memphis/31/98, was inhibited by 1 μM oseltamivir, with barely detectable HA titer and little cytopathic effect (cpe) in the cultures. A/OK/03 replication was not inhibited up to 10 μM oseltamivir with little change in virus yield and high cpe. A/OK/309/06 consistently showed higher virus yield as oseltamivir concentration was increased from 1 nM to 1 μM (Figure 5).

We passaged A/Oklahoma/323/03 and A/Oklahoma/309/06 three times in the presence of 10 μM oseltamivir. RT-PCR of viral RNA from the last passage showed no trace of the full length NA band and very weak shorter products (Figure 5B). We sequenced some short bands from each virus and found multiple junctions in A/Oklahoma/309/06 similar to those seen after passage without oseltamivir (Table 2). A/Oklahoma/323/03 contained a long internal deletion (192–1209) but also showed two other small deletions (1288–1300 and 1412–1418, Table 2).

### Deletions are not randomly occurring in other gene segments

The PCR products shown in Figure 3 were obtained using NA-specific primers, and all the sequences we obtained of subgenomic PCR products were derived from the NA gene segment. Growth of influenza at high multiplicity is known to generate defective (DI) particles containing internally deleted fragments of RNA segments, particularly from the polymerase genes. Therefore it was possible that the deletions we found in NA merely reflected increasing proportions of randomly generated DI viruses. To investigate if the deletions were occurring in all RNA segments we amplified RNA from selected passages by RT-PCR using common primers that will amplify all 8 segments of influenza virus. There were several small bands obtained from the universal primers in addition to those amplified by NA-specific primers but sequences obtained indicated that most were the result of mis-priming. We found only one internal deletion from another gene, which was an internal deletion of 1394 nucleotides from segment 5 RNA, coding for the NP. We conclude that there were few deletions occurring in the other gene segments and that the NA was specifically lost when A/OK/323/03 was passaged at an moi of ~0.01.

### Properties and sequence of the HA did not change when NA gene was deleted

The NA-deficient virus stock of A/OK/323/03 shown in Figure 1 yielded no visible full-length NA PCR product and has only background NA activity. To see if the loss of requirement for NA activity was accompanied by a change in HA, we sequenced the HA segment in virus stocks that showed no full length NA segment. There were no changes in the HA sequences when compared to HA of viruses passaged at low multiplicity.

### HAs of 2003–2008 H3N2 viruses have different receptor specificities

**Figure 6:**
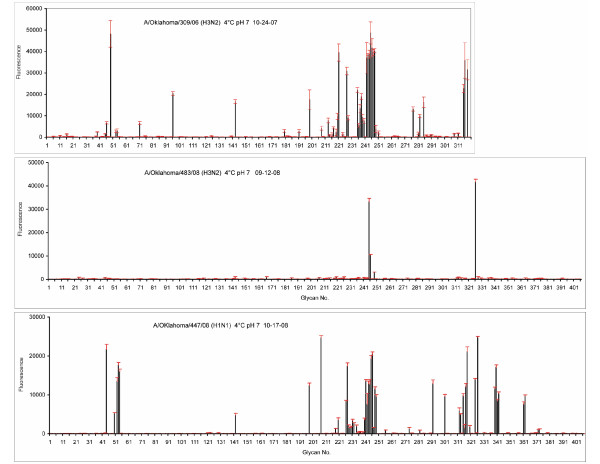
**Glycan Array results for A/OK/309/06 and A/OK/483/08**. A/OK/309/06 (top panel) was run on CFG Printed Array version 3.0 (320 glycans) while the others are v3.2 (406 glycans). For comparison, results are also shown for an H1N1 2008 isolate that binds to nearly every α2–6 sialylated glycan on the array (bottom panel). The array keys and data are available on the CFG web site [32]. The results are summarized in Table 3.

One mechanism of resistance to NA inhibitors is by mutations in the HA that lower avidity for receptors, thus allowing the virus to shake itself off receptors without the need for NA activity. There was no change in the HA sequence as the viruses were passaged, even when the full length NA gene became lost after multiple passages of A/OK/323/03. However, we wondered if there were intrinsic differences in HA binding in those viruses that are susceptible to loss of NA when grown in MDCK cells. We previously showed that there were only quantitative differences in binding specificity when viruses from 2003 and 2005 were assayed on the Glycan Array of the Consortium for Functional Glycomics, even though the viruses showed varying ability to agglutinate chicken red cells and one virus had been adapted to grow in embryonated chicken eggs [9]. We labeled purified viruses with Alexa 488 under conditions that did not alter hemagglutination titers and tested binding to current versions of the Glycan Array (Figure 6). The Wisconsin-like virus A/OK/309/06 binds more glycans than the Fujian/02 and California/04 groups of viruses. In addition to the minimal motif bound by those viruses [9], A/OK/309/06 bound to a trisaccharide containing 9-O-acetyl-N-acetyl neuraminic acid, and also to any glycan with sialic acid linked α2–6 to N-acetylgalactosamine, including when this disaccharide forms an internal branch. In marked contrast, the Brisbane-like isolate A/OK/483/08 bound only when the sialic acid was linked 2–6 to an extended polylactosamine. There was no change in this pattern when different concentrations of virus were tested or when re-tested a month later with a different preparation of Alexa-labeled virus. Another Brisbane-like H3N2 isolate, A/OK/1123/08, showed the same restricted binding specificity. The minimal units bound by these viruses are shown in Table 3. As a control for the binding experiment, an H1N1 isolate from 2008 bound to essentially every 2–6 sialylated glycan on the array, which by then contained 406 glycans (Figure 6).

**Table 3:** Minimal oligosaccharides bound by recent H3N2 viruses

Virus	Minimal binding motifs
A/OK/323/03 (Fujian-like)^1^	Neu5Acα2–6Galβ1–4GlcNAc Neu5Acα2–6GalNAcβ1–4GlcNAc

A/OK/1992/05 (California-like)^1^	Neu5Acα2–6Galβ1–4GlcNAc Neu5Acα2–6GalNAcβ1–4GlcNAc

A/OK/309/06 (Wisconsin-like)	Neu5Acα2–6Galβ1–4GlcNAc Neu5Acα2–6GalNAcβ1–4GlcNAc 9OAcNeu5Acα2–6Galβ1–4GlcNAc Neu5Acα2–6GalNAc in any context

A/OK/483/08 (Brisbane-like)	Neu5Acα2–6Galβ1–4GlcNAcβ1–3Galβ1–4GlcNAc

^1^Data previously reported [9]

### Secondary structures in NA gene segments

**Figure 7:**
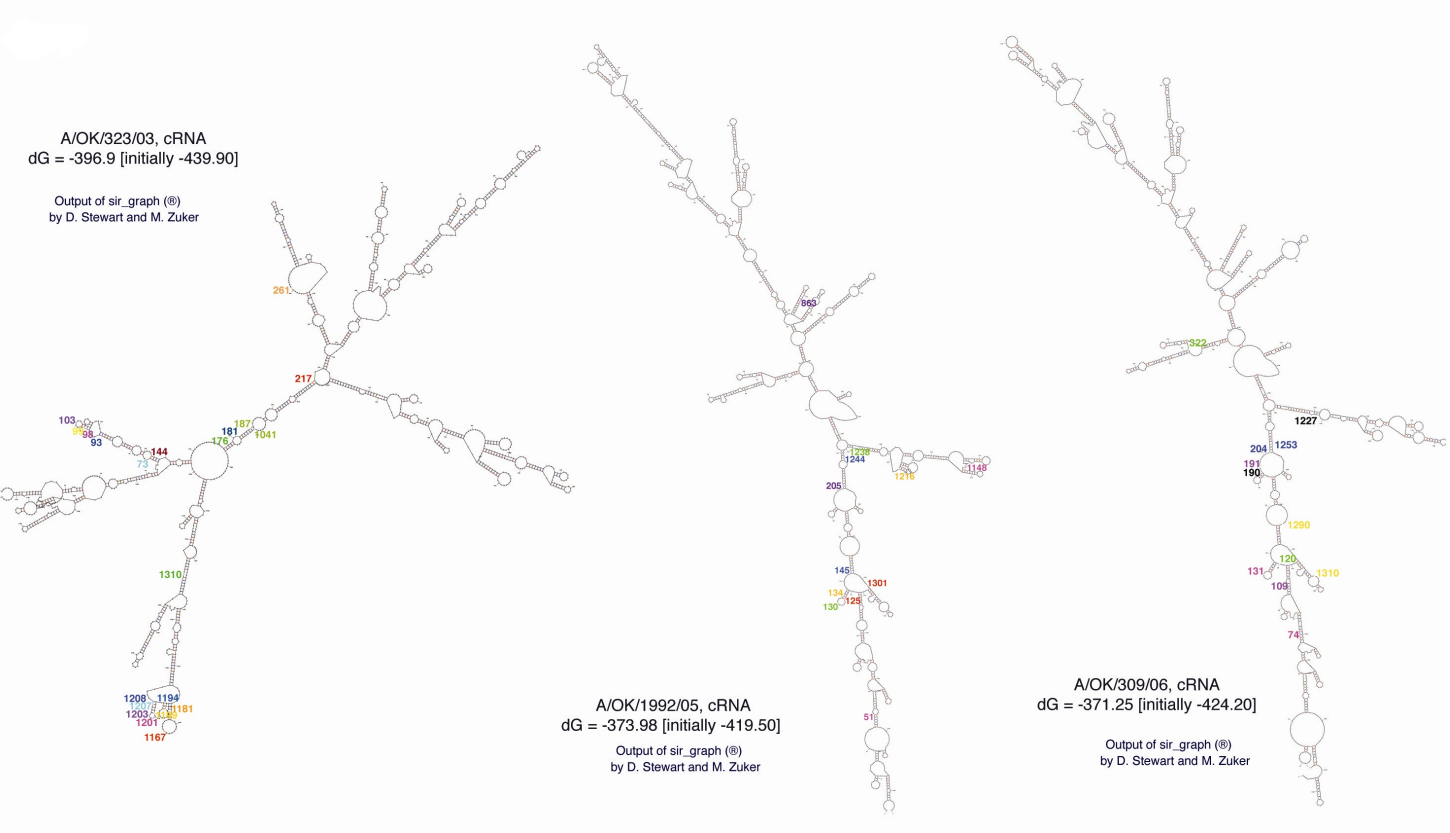
**Potential secondary structures in RNA segment 6 for A/OK/323/03 (left), A/OK/1992/05 (center) and A/OK/309/06 (right)**. Results shown are for the positive (sense) strand and were generated by the mFold RNA folding program [10,11]. The lowest energy foldings are shown. All are the positive strand; A/OK/1992/05 and A/OK/309/06 were obtained using default parameters in mFold, while A/OK/323/03 was obtained using the adjusted parameters listed in the Methods. Nucleotides at junctions in the deleted segments are shown, color coded by nucleotides that come together when the internal section is deleted.

To investigate if the secondary structure of RNA plays a role in generating internal deletions, we examined possible secondary folding of the NA gene (RNA segment 6) of the Oklahoma viruses using the program mFold version 3.2 [10,11]. We ran the program with both negative sense viral RNA (vRNA) and the positive sense full length RNA (cRNA) strands of A/OK/323/03, A/OK/1992/05 and A/OK/309/06, using default RNA parameters or with varied parameters. All the NA genes showed extensive secondary structures with only slight variations as the parameters were changed. Notably, all lower energy structures brought the 5' and 3' ends into close proximity. The most probable structures in the mFold output using default parameters are shown in Figure 7, with the positions of junctions in the deleted segments color coded (e.g. red is joined to red). In some cases, the nucleotides that form the junction when the segment is deleted are juxtaposed in the predicted secondary structure (187/1041 in A/OK/323/03, 125/1301 in A/OK/1992/05, 204/1253 in A/OK/309/06). In other cases, folding stem-containing arms into a tertiary structure would bring groups of junctions together (e.g. 93–103 to 1194–1208 in A/OK/323/03, 51, 134, 145 to 1148, 1216, 1244 in A/OK/1992/05). Note that in some cases there are ambiguities in assigning junctions due to repeated nucleotides; for example, the junction listed as 80/1289 in Table 2 is actually somewhere in the range 77/1286 to 81/1290. In other cases the ambiguity is 1–2 nucleotides but in many junctions there is no overlap so the assignment is precise. While the details are slightly different when the negative sense vRNAs are folded, in all cases we observed a predominance of junctions in loops or close to the ends of hairpins. These results suggest that deletions occur by polymerase skipping across the ends of hairpins in secondary and tertiary structure and they could originate on either positive or negative sense RNA.

## Discussion

During influenza infection, viruses that lack NA activity usually fail to spread to new cells because they aggregate at the infected cell surface due to binding of the HA to sialic acid on the surface glycoproteins of neighboring virus particles [1,2]. We previously isolated a mutant of NWS-G70c (H1N9) virus that lacked NA activity due to a large internal deletion in the NA gene, but this virus was selected by adding bacterial sialidase to the growth medium and was dependent on exogenous sialidase for multicycle growth [12]. The low-NA H3N2 viruses reported here do not require added sialidase for efficient growth in MDCK cells. The viruses isolated in 2003, 2005 and 2006 have different susceptibilities to deletions in the NA segment. A/OK/323/03 retained full NA segment length and full NA activity when passaged strictly under limiting dilution conditions (moi ~10^-5^), but generated internal deletions of the NA gene when passaged at higher moi of 0.01, with different deletions at each passage. A/OK/1992/05 generated deleted NA segments that increased with passage number, but there was only slight difference if the passages were made at limiting dilution or at 0.01 moi. A/OK/309/06 and A/OK/483/08 showed deletions even after a single passage in MDCK cells. The 5' and 3' ends were always retained, in accord with these being the location of specific packaging signals [13,14]. We were unable to plaque-purify viruses with deleted NA gene; there was always full length NA present. This suggested there was some advantage of either full length NA gene or NA activity. All these viruses were resistant to oseltamivir in replication assays but the NA was sensitive in the enzyme assay, suggesting NA activity is not required and therefore the requirement might be for the full-length NA-coding RNA segment.

### The deletions are targeted to the NA gene segment

The PCR reactions in Figures 1 and 3 were carried out with NA-specific primers, so there was a possibility that the deletions we saw in NA were just a subset of general genome-wide deletions. It is well known that high-multiplicity passage of influenza virus leads to accumulation of defective-interfering (DI) particles containing sub-genomic RNAs resulting from internal deletions, most commonly in the polymerase gene segments [15-19]. We looked for subgenomic RNAs derived from other gene segments by using PCR primers with the common 3' and 5' gene segment sequences. We found one non-NA subgenomic RNA with an internal deletion in the NP RNA segment. Other sub-genomic bands resulted from mis-priming, presumably due to the short sequences (12 nt and 13 nt) used to start the amplification. We conclude that the large number of NA deletions we found result from lack of selective pressure to retain NA when these viruses replicate in MDCK cells.

### Do H3N2 viruses delete NA because they have low receptor affinity or narrow specificity?

The 2003–2008 viruses used for these experiments all agglutinate human red blood cells with a similar avidity, giving titers of 16–64 when grown in MDCK cells. The specific HA titers of purified virus preparations were not significantly different (3–4 log_2_HAU per μg viral protein). However, glycan array analysis showed different specificities. A/OK/309/06 had the broadest specificity, followed by the 2003–5 viruses, while A/OK/483/08 was restricted in its binding to sialylated polylactosamines of at least 5 sugars (Figure 6 and Table 3). There was no correlation between the variety of glycans bound and the ease of generating deletions in NA. The signal strength is similar when similar amounts of virus are applied to the array. If the diversity of glycans on the surface of an MDCK cell is similar to that on the array we might expect that affinity, and NA dependence, would correlate with diversity of binding, but there is little information on the glycans present on MDCK cells. The greatest loss in NA was seen in A/OK/323/03, which is intermediate in binding specificity. The HA sequence did not change in viruses as they deleted the NA segment.

### NA is not always a receptor-destroying enzyme

If the only function of NA is to cleave receptors bound by the HA, then HA and NA activity of a viable virus should be matched quantitatively (Kd of HA and Kcat of NA) and qualitatively (same specificity). Several studies have found that lower NA activity can be compensated by low HA avidity [4-7,20,21] leading to conclusions that the activities are balanced. However, these studies did not consider specificity of binding and release. Many recent H3N2 viruses do not elute from red cells by their NA activity, showing that the specificities of HA and NA are mismatched [8]. The NA activity of A/OK/323/03 can cleave α2–6-linked sialic acid from the trisaccharide sialyllactose but does not release the virus from red cells, indicating that the HA binds to high affinity ligands that have a structure that is resistant to viral NA activity but sensitive to sialidase from *M. viridifaciens *[8]. The hemagglutinating site on N9 NA shows similar resistance to cleavage by viral NA activity [22].

Oseltamivir inhibits the NA activity of A/OK/323/03 and A/OK/309/06 but does not inhibit virus growth in MDCK cells (Figure 5). Mechanisms that allow virus propagation in the presence of NA inhibitors include mutations in the drug binding site of NA, as in the resistance of recent H1N1 human isolates that carry the H274Y mutation, but in the 2003–2008 viruses studied here the NA enzyme activity is fully sensitive to oseltamivir inhibition. A/OK/309/06 was resistant to oseltamivir after a single passage in MDCK cells, suggesting the potential exists for resistance in humans by a mechanism in which NA activity is not required. It has been noted that aggregation of virus particles seen in electron micrographs when influenza virus is grown without NA activity is due to virus-virus interactions rather than the virus-cell interactions [1,2] and it seems likely that it is more important for NA to cleave sialic acids from the viral glycoproteins (only N-linked glycans) than from the cell surface (N-linked and O-linked glycans and glycolipids). Thus the specificities of HA and NA do not need to be matched and, furthermore, NA is not required if the HA does not bind to sialic acid structures on other HAs. NAs of human viruses have a marked preference for α2–3 linked sialic acid, in contrast to the strict requirement of HA for α2–6 sialylated glycans.

We previously showed that a reassortant H1N9 virus, NWS-G70c, progressively lost NA coding capacity when passaged in the presence of bacterial sialidase and anti-NA antiserum [2,12,23]. The result was a group of viruses that had no full-length NA segment and therefore no coding capacity for the active NA enzyme. Each virus had a single internal deletion in the NA gene with retention of at least 100 nucleotides at the 3' end of the genomic RNA and 200 nucleotides at the 5' end [23]. These viruses were dependent on added sialidase for multi-cycle replication and we did not succeed in adapting them to grow without exogenous sialidase. Kawaoka and colleagues replicated these NWS-G70c NA deletions by passaging the virus in MDCK cells that constitutively expressed N2 NA. The resulting NA-deficient virus was gradually weaned off its sialidase requirement and eventually variants were obtained that could grow without added sialidase in MDCK cells, eggs or mice. These viruses had low or zero ability to agglutinate chicken red cells and they had multiple mutations in the HA gene [6]. The authors concluded that adaptation to grow without NA activity required a reduction in receptor binding as occurs in NA inhibitor-resistant mutants that can be released from their receptors by thermal motion and so are not dependent on NA activity [24-27].

Deletions in NA but with no change in HA were reported by Hughes et al. [7] when an H3N2 virus, A/Tottori/872/94, was passaged in MDCK cells that had reduced surface sialic acid. The authors concluded that the lower density of sialic acids on the cell surface and on the viral glycoproteins resulted in lower avidity, thus allowing virus spread in the absence of NA activity. However, the infectivity was reduced by several orders of magnitude.

The NA-deficient stocks of A/OK/323/03 reported here have very different properties. They were generated by passaging virus in normal MDCK cells without any exogenous sialidase. There was no decrease in infectious titer during these passages and no change in HA activity. Ferraris et al. [28] also reported H3N2 isolates from 2002 to 2005 that had no detectable NA activity. The 2003–2004 isolates that were further studied were resistant to NA inhibitors and yielded no full-length NA PCR product. The properties that we have described may be common among recent H3N2 viruses. The concern is that these viruses can replicate in the absence (or near absence) of NA activity, and so are resistant to oseltamivir (Figure 5). The OK/323/03 virus only acquired this trait after several passages in MDCK cells, but the other viruses were resistant after a single passage.

### What is the selection for full length NA RNA?

The full-length NA gene segment of A/OK/323/03 was never completely lost and we were unable to plaque-purify a virus with no full-length NA segment. This is in accord with the observation that different deletions were found at each passage (Figure 4), suggesting the deletions were generated anew during each passage.

It is important to note that the two conditions of passage used for A/OK/323/03 were not strikingly different. While one was strictly limiting dilution, the "high-multiplicity" passages were inoculated with an moi of about 0.01. We saw no accumulation of deleted polymerase gene fragments, as are routinely found in infections when the moi is >1 [18,19]. Cairns and Fazakas noted many years ago that if cells are infected at high multiplicity, the virus yield is much lower than if the infection is initiated with one infectious particle, despite the fact that the final cycle of a multi-cycle replication from a single infectious unit occurs at high multiplicity [29]. They also pointed out that the total yield of virus at the end of a multi-cycle replication has an upper limit that is determined by the number of cells, not by the number of infectious particles that initiated the infection, except when the infection is poor due to a high moi. We found that the virus yield, whether measured as infectious units (TCIU) or as particles (HA titration), is the same from 12 passages under 0.01-moi conditions (1000 TCIU per well) as from limiting dilution (~3 TCIU). Therefore replication is not impaired by the higher moi or by the loss of NA activity. Our results could be interpreted either as a positive selection for shorter NA segments, with little or no requirement for NA activity, or as negative selection against NA activity but a requirement for at least the 3' and 5' NA RNA sequences. The total yield of virus per well (10^5 ^cells) was 2–3 × 10^6 ^TCIUs, so if NA deletions occur at a frequency of 10^-4 ^or 10^-5^, positive or negative selection may occur during higher multiplicity multi-cycle infections but would not be observed at limiting dilution.

## Conclusion

The mechanism of deletion of internal segments of NA requires two parts; a loss of selective pressure to retain NA activity, and a mechanism to generate the deletions. Winter et al proposed that influenza polymerase can generate deletions by jumping across the ends of hairpins and even to other templates [17,30] and the secondary structure plots generated by mFold (Figure 7) support this idea for the NA deletions, with tertiary structures also involved. The loss of NA, and the ability of the human H3N2 viruses to grow in the presence of an NA inhibitor, is not accompanied by changes in HA and appears to be a new mechanism of resistance to NA inhibitors; lack of requirement for NA activity as a consequence of a lack of binding of HA to sialylated glycans on the viral glycoproteins.

It seems likely that the retention of at least a trace full-length NA RNA segment in our experiments is due to its benefits in packaging rather than the low amount of NA activity that would be provided. The results may be best explained if virus that has packaged eight full-length RNAs is preferentially infectious but undergoes deletion during replication because NA activity is not required.

## Methods

### Viruses and cells

The viruses used in this study were A/Oklahoma/323/03, a Fujian-like isolate (H3N2), [8], A/Oklahoma/1992/05, a California-like H3N2 isolate, A/Oklahoma/309/06, a Wisconsin-like H3N2 isolate and A/NWS/33(P227H)_HA _– A/Memphis/31/98_NA _(NWS-Mem/31/98, H1N2, [23]). Viruses were grown in Madin-Darby canine kidney (MDCK) cells in DMEM:Ham's F12 medium (1:1) with ITS+ (BD Biosciences) and trypsin added as previously described [12]. For RNA extraction, the virus-containing medium was cleared of cell debris (3,000 g for 5 min) then virus was concentrated by sedimentation (SW28 rotor, 25,000 rpm for 2 hr at 4°). The virus pellet was resuspended in CaMg-saline (0.25 mM CaCl_2_, 0.8 mM MgCl_2 _in borate buffered saline, pH 7.2).

### Isolation of viral RNA, reverse transcription, and PCR amplification (RT-PCR)

Viral RNA was isolated from the pelleted virus using the QiAmp Viral RNA extraction mini kit (Qiagen). cDNA was synthesized using the Omniscript RT kit (Qiagen) and an oligodeoxynucleotide (5'-AGCAAAAGCAGG) that is complementary to the 12 conserved nucleotides at the 3' end of all influenza type A viral RNA segments. To amplify the N2 NA fragment a pair of N2 NA specific primers were used: 5'-GGGTCGACGCGTTTG**AGCAAAAGCAGGAGTGAAAAT**, complementary to nucleotides 1–21 at the 3' end of viral RNA (bolded) preceded by a T3 promoter sequence, and 5'-CGGAATTCATTAACCCTCACTAAA**AGTAGAAACAAGGAG **for the 5' end. The PCR conditions were 94° for 5 min, then 5 cycles of: 94° for 1 min, anneal at 32° for 1 min, extend at 72° for 2 min, followed by 25 cycles at higher temperature for specific binding of NA primers (94° 1 min, anneal at 68° 1 min, synthesis at 72° for 2 min and finally 72° for 7 min). To amplify all the 8 segments of influenza virus we used a pair of universal primers, containing the 12 and 13 nucleotides complementary to the 3' and 5' terminal sequences of all 8 RNA segments, attached to T7 promoter sequences for additional length. The primers were 5'-AATACGACTCACTATA**AGCAAAAGCAGG**, complementary to the 3' end of viral RNA and 5'-CGGAATTCAATACGACTCACTATA**AGTAGAAACAAGG **for the 5' end. The PCR conditions were 94° for 20 sec, anneal at 30° for 30 sec, extend at 72° for 5 min for 3 cycles followed by 30 cycles of 94° 20 sec, anneal at 55° for 30 sec, extend at 72° for 15 min, and finally 72° 7 min. The PCR products were separated by electrophoresis on a 1% agarose gel and extracted using the QiA Quick Gel Extraction kit (Qiagen). The purified RT-PCR products were then sequenced in the Oklahoma Medical Research Foundation DNA Sequencing Facility using an ABI 3730 Capillary Sequencer with the NA specific primers for NA or universal primers for the 8 segments.

### Hemagglutination and neuraminidase assays

Hemagglutinin titrations were done in 96 well plates using 50 μl of serial dilutions of virus harvested from MDCK cells, and adding 50 μl of 0.8% human red blood cells. The plate was kept at 4°C and agglutination was read at 90 min. Neuraminidase assays were done by fluorescence using 4-methylumbelliferylα-N-acetylneuraminic acid as substrate [31].

### NA/HA ratios and tissue culture infectious units (TCIU)

To relate the NA activity to virus amount we used a ratio of NA to HA. For NA, this was the fluorescence reading generated by 5 μl virus-containing medium in 15 min divided by the log_2 _of the HA titer of 50 μl of virus. This arbitrary scale allowed comparison with the log(TCIU). One TCIU was estimated as the geometric mean of the last well showing infection in the 10-fold dilution series and the next well. The assays were done immediately after harvesting the virus in MDCK supernatants to minimize any instability in either HA or NA.

### Virus growth in the presence of oseltamivir carboxylate

Viruses as serial 10-fold dilutions were used to infect MDCK cells in the absence or presence of 10 μM to 0.001 μM oseltamivir added to the infection medium. After 3 days incubation at 37°C the cytopathic effect (cpe) was estimated by eye and the HA titer was determined.

### Cloning of deleted NA fragments of A/Oklahoma/309/06

We ligated the gel band at about 0.7 kb into pBluescript IIKS^+ ^digested with EcoRV and transformed it into One-shot Top10 competent cells (Invitrogen). Three clones were sequenced using primers T7 Promoter 5' TAATACGACTCACTATAGGG complementary to the 3' end and M13 5' AACAGCTATGACCAT for the 5' end, as well as M13 5' GTTTTCCCAGTCACGAC complementary to 3' end and M13 5' CAGGAAACAGCTATGAC for the 5' end.

### Glycan Array analysis

Viruses were purified by 5–20% sucrose gradient centrifugation and the purification checked by SDS gel electrophoresis. Labeling with Alexa-488 succinimidyl ester (Molecular Probes) was monitored by HA titration and a level chosen that did not result in reduction of HA titer; for these viruses 0.005 μg Alexa per HAU. The reaction conditions were as previously described [9]. After dialysis the samples contained about 50 log_2_HAU per ml and about 1 mg/ml viral protein. The Glycan Arrays printed on glass slides were run by Core H of the Consortium for Functional Glycomics. Alexa-labeled viruses were diluted empirically and the slides incubated for 1 hour at pH 7, 4°C then washed and fluorescence measured using the standard buffers and procedures of Core H [32]. Increasing concentrations of virus were applied to the same slide so approximate binding curves could be plotted for each glycan.

### RNA secondary structure analysis

The program mFold [10,11] was used to probe for possible secondary structures in RNA segment 6. We compared the results using the default parameters and with the following changes: Percent suboptimality 10 (default 5), Maximum interior/bulge loop size and maximum asymmetry of bulge/loop both 10 (default 30).

## Competing interests

The authors declare that they have no competing interests.

## Authors' contributions

SG carried out the experiments, contributed to their design and drafted the manuscript. DFS helped design the Glycan Array experiments, oversaw the array analyses and assisted with their interpretation. GMA conceived the study, contributed to experimental design, interpreted the results and completed the manuscript. All authors read and approved the final manuscript.

## References

[B1] Palese P, Tobita K, Ueda M, Compans RW (1974). Characterization of temperature-sensitive influenza virus mutants defective in neuraminidase. Virology.

[B2] Liu C, Eichelberger MC, Compans RW, Air GM (1995). Influenza type A virus neuraminidase does not play a role in viral entry, replication, assembly, or budding. J Virol.

[B3] Baum LG, Paulson JC (1991). The N2 neuraminidase of human influenza virus has acquired a substrate specificity complementary to the hemagglutinin receptor specificity. Virology.

[B4] Mitnaul LJ, Matrosovich MN, Castrucci MR, Tuzikov AB, Bovin NV, Kobasa D, Kawaoka Y (2000). Balanced hemagglutinin and neuraminidase activities are critical for efficient replication of influenza A virus. J Virol.

[B5] Wagner R, Matrosovich M, Klenk HD (2002). Functional balance between haemagglutinin and neuraminidase in influenza virus infections. Rev Med Virol.

[B6] Hughes MT, Matrosovich M, Rodgers ME, McGregor M, Kawaoka Y (2000). Influenza A viruses lacking sialidase activity can undergo multiple cycles of replication in cell culture, eggs, or mice. J Virol.

[B7] Hughes MT, McGregor M, Suzuki T, Suzuki Y, Kawaoka Y (2001). Adaptation of influenza A viruses to cells expressing low levels of sialic acid leads to loss of neuraminidase activity. J Virol.

[B8] Gulati U, Wu W, Gulati S, Kumari K, Waner JL, Air GM (2005). Mismatched hemagglutinin and neuraminidase specificities in recent human H3N2 influenza viruses. Virology.

[B9] Kumari K, Gulati S, Smith DF, Gulati U, Cummings RD, Air GM (2007). Receptor binding specificity of recent human H3N2 influenza viruses. Virol J.

[B10] Mathews DH, Turner DH, Zuker M (2007). RNA secondary structure prediction. Curr Protoc Nucleic Acid Chem.

[B11] Zuker M (2003). Mfold web server for nucleic acid folding and hybridization prediction. Nucleic Acids Res.

[B12] Liu C, Air GM (1993). Selection and characterization of a neuraminidase-minus mutant of influenza virus and its rescue by cloned neuraminidase genes. Virology.

[B13] Fujii K, Fujii Y, Noda T, Muramoto Y, Watanabe T, Takada A, Goto H, Horimoto T, Kawaoka Y (2005). Importance of both the coding and the segment-specific noncoding regions of the influenza A virus NS segment for its efficient incorporation into virions. J Virol.

[B14] Fujii Y, Goto H, Watanabe T, Yoshida T, Kawaoka Y (2003). Selective incorporation of influenza virus RNA segments into virions. Proc Natl Acad Sci USA.

[B15] Odagiri T, Tashiro M (1997). Segment-specific noncoding sequences of the influenza virus genome RNA are involved in the specific competition between defective interfering RNA and its progenitor RNA segment at the virion assembly step. J Virol.

[B16] Chambers TM, Webster RG (1987). Defective interfering virus associated with A/Chicken/Pennsylvania/83 influenza virus. J Virol.

[B17] Jennings PA, Finch JT, Winter G, Robertson JS (1983). Does the higher order structure of the influenza virus ribonucleoprotein guide sequence rearrangements in influenza viral RNA?. Cell.

[B18] Kantorovich-Prokudina EN, Semyonova NP, Berezina ON, Zhdanov VM (1980). Gradual changes of influenza virions during passage of undiluted material. J Gen Virol.

[B19] Davis AR, Hiti AL, Nayak DP (1980). Influenza defective interfering viral RNA is formed by internal deletion of genomic RNA. Proc Natl Acad Sci USA.

[B20] Kaverin NV, Gambaryan AS, Bovin NV, Rudneva IA, Shilov AA, Khodova OM, Varich NL, Sinitsin BV, Makarova NV, Kropotkina EA (1998). Postreassortment changes in influenza A virus hemagglutinin restoring HA-NA functional match. Virology.

[B21] Lu B, Zhou H, Ye D, Kemble G, Jin H (2005). Improvement of influenza A/Fujian/411/02 (H3N2) virus growth in embryonated chicken eggs by balancing the hemagglutinin and neuraminidase activities, using reverse genetics. J Virol.

[B22] Air GM, Laver WG (1995). Red cells bound to influenza virus N9 neuraminidase are not released by the N9 neuraminidase activity. Virology.

[B23] Yang P, Bansal A, Liu C, Air GM (1997). Hemagglutinin specificity and neuraminidase coding capacity of neuraminidase-deficient influenza viruses. Virology.

[B24] Bantia S, Ghate AA, Ananth SL, Babu YS, Air GM, Walsh GM (1998). Generation and characterization of a mutant of influenza A virus selected with the neuraminidase inhibitor BCX-140. Antimicrob Agents Chemother.

[B25] Gubareva LV (2004). Molecular mechanisms of influenza virus resistance to neuraminidase inhibitors. Virus Res.

[B26] McKimm-Breschkin JL (2000). Resistance of influenza viruses to neuraminidase inhibitors – a review. Antiviral Res.

[B27] Staschke KA, Colacino JM, Baxter AJ, Air GM, Bansal A, Hornback WJ, Munroe JE, Laver WG (1995). Molecular basis for the resistance of influenza viruses to 4-guanidino-Neu5Ac2en. Virology.

[B28] Ferraris O, Kessler N, Valette M, Lina B (2006). Evolution of the susceptibility to antiviral drugs of A/H3N2 influenza viruses isolated in France from 2002 to 2005. Vaccine.

[B29] Cairns HJF, Fazekas de St Groth S, Edney M (1952). Quantitative aspects of influenza virus multiplication. J Immunol.

[B30] Fields S, Winter G (1982). Nucleotide sequences of influenza virus segments 1 and 3 reveal mosaic structure of a small viral RNA segment. Cell.

[B31] Potier M, Mameli L, Bélisle M, Dallaire L, Melançon SB (1979). Fluorometric assay of neuraminidase with a sodium (4-methylumbelliferyl)-α-D-N-acetylneuraminate substrate. Anal Biochem.

[B32] Consortium for Functional Glycomics. http://www.functionalglycomics.org/static/consortium/resources/resourcecoreh8.shtml.

